# Inpatient versus outpatient management of community-acquired acute skin and soft tissue infections. Clinical outcomes and factors associated with eligibility for early discharge

**DOI:** 10.1186/s12879-025-11883-6

**Published:** 2025-11-17

**Authors:** Elena Sendra, Inmaculada López-Montesinos, Estela Membrilla-Fernández, Ana María Gonzalez-Castillo, Francisca Sánchez, Esperanza Cañas-Ruano, Silvia Castañeda, Judith Poblet-Florentin, Rosana Sabaté, Alicia Rodríguez-Alarcón, Sandra Esteban-Cucó, Amaya Suárez, Soukaina Sara Alanti, Xavier Duran-Jordà, Adrián Vizoso-Expósito, Silvia Gómez-Zorrilla, Juan Pablo Horcajada

**Affiliations:** 1 Infectious Diseases Service, Hospital del Mar. Hospital del Mar Research Institute, Passeig Marítim 25-27, Barcelona, 08003 Spain; 2 Universitat Autònoma de Barcelona (UAB), Barcelona, Spain; 3 Universitat Pompeu Fabra (UPF), Barcelona, Spain; 4 CIBER of Infectious Diseases (CIBERINFEC), Health Institute Carlos III, Madrid, Spain; 5 General Surgery Service, Hospital del Mar. Hospital del Mar Research Institute, Barcelona, Spain; 6 Internal Medicine Service, Hospital del Mar. Hospital del Mar Research Institute, Barcelona, Spain; 7 Pharmacy Service, Hospital del Mar, Hospital del Mar Research Institute, Barcelona, Spain; 8 Microbiology Service, Laboratori de Referència de Catalunya, Hospital del Mar, Barcelona, Spain; 9 Methodology and Biostatistics Support Unit, Hospital del Mar Research Institute, Barcelona, Spain

**Keywords:** Skin and soft tissue infections (SSTIs), Cellulitis, Outpatient care, Antimicrobial stewardship, Antibiotic, Switch -to-oral therapy

## Abstract

**Background:**

Skin and soft tissue infections (SSTIs) cause an increasing demand for inpatient and outpatient medical care. In recent years, health systems have promoted strategies to treat selected patients on an outpatient basis, which may avoid hospital admissions and associated complications.

**Methods:**

A retrospective, cohort study of adults presenting to the emergency department (ED) with a SSTI between 2018 and 2020. Primary objective: to compare clinical outcomes in patients with SSTI treated as outpatients or inpatients. Primary outcome: Early clinical failure. Secondary outcomes: Recurrences, unplanned readmissions and ED visits related to the SSTI. Secondary objective: to investigate factors associated with eligibility for early discharge of inpatients. Logistic regression analysis was used to control for confounding factors.

**Results:**

Three hundred twenty patients were included, 160 hospitalized. The median hospital stay was 9 days (IQR 5–15), and 20 patients (12.5%) were discharged within 72 h after admission. In multivariate analysis, no differences were observed between groups in rates of early clinical failure, recurrences and unplanned readmissions. Hospitalized patients were significantly less likely to revisit the emergency department for SSTI-related issues (OR 0.32, 95% CI 0.16–0.62; *p* = 0.001). Social and economic barriers to medical care were associated with lower probability of being eligible for early discharge (OR 0.19 95% CI 0.07–0.54; *p* = 0.002). The severity of the infection was associated with failure to meet the eligibility criteria for early discharge (OR 0.92 95% CI 0.88–0.96; *p* < 0.001).

**Conclusions:**

There were no differences in early clinical failure between admitted and non-admitted patients. Social and economic barriers to medical care and severity of infection were associated with not fulfilling the criteria for eligibility for early discharge.

**Supplementary Information:**

The online version contains supplementary material available at 10.1186/s12879-025-11883-6.

## Introduction

Bacterial skin and soft tissue infections (SSTIs) are currently one of the most common reasons for emergency department (ED) visits and subsequent hospital admissions [1]. Studies have reported an increase in the incidence and economic burden of SSTIs in recent years [2, 3]. One of the reasons for this increase is the emergence of community-associated methicillin-resistant *Staphylococcus aureus* (MRSA) in recent decades [4].

SSTIs are often associated with susceptible populations, such as the elderly, patients with comorbidities, persons who inject drugs (PWID) or are experiencing homelessness [5, 6]. Ambulatory approaches based on outpatient parenteral antimicrobial therapy (OPAT) programs and oral antibiotic switch therapy represent effective alternatives to hospitalization in these patients [7, 8] The recent development of long-acting lipoglycopeptides may be useful in the outpatient management of SSTIs due to their broad activity against Gram- positive cocci (GPC) and long half-lives [9], and particularly useful in patients for whom antibiotic treatment adherence or follow-up is difficult, such as PWID [10].

In recent years, healthcare systems have encouraged ambulatory care practices to reduce the healthcare burden associated with hospitalization. However, few previous studies have set out to analyse the possible predictors that can help identify the subset of patients who may benefit from management in the outpatient setting, based on their risk of clinical failure and/or death [11–14].

We hypothesized that outpatient management is not-inferior to hospitalization in selected patients with community-acquired SSTIs. This study aims to compare differences in clinical outcomes between inpatient and outpatient management of community-acquired SSTIs. The secondary objective is to investigate factors associated with suitability for early discharge in hospitalized patients.

## Methods

### Setting, study design, and study population

A retrospective, cohort study was conducted from January 2018 to December 2020 at the Hospital del Mar, a 450-bed, tertiary care teaching hospital in Barcelona (Spain). Episodes of SSTIs were identified from the hospital’s documentation service and pertinent data were retrieved from hospital charts, using data from January 2018 to May 2021. All consecutive adult patients aged 18 years or older who presented to the ED with a diagnosis of community-acquired SSTI, including cellulitis/erysipelas, wound infections, necrotizing fasciitis and skin abscesses, were retrospectively reviewed. Patients were including consecutively, starting from the initial data of the study (January 2018) until the required sample size was achieved. Hospital-acquired infections, mild SSTIs, patients with other infections at the time of admission, patients lost to follow-up and patients with a diagnosis of paronychia, pustulosis, lymphangitis, lymphadenitis, boils, carbuncles, impetigo, epidermoid cyst or pilonidal cyst without abscess were excluded. Mild SSTIs were considered when patients did not present with systemic involvement (such as altered mental status, fever, hypotension, or tachycardia), or when no laboratory tests were available to assess systemic involvement through elevated parameters, such as leukocytosis or increased acute-phase reactants (e.g., C-reactive protein or procalcitonin). Outpatient evolution was evaluated during outpatient visits to the hospital and primary care centres by reviewing the patient’s medical records through the electronic health record system of the Catalonia healthcare region. The comprehensive nature of the electronic system allowed for consistent monitoring of outpatient visits and facilitated an in-depth retrospective evaluation of patient outcomes. Patients were followed for up to 30 days after the date of discharge from the hospital. In cases of more than one SSTI episode, the second episode was assessed only if it occurred at least 30 days after discharge from the previous episode and if the first episode had previously resolved.

This article was written according to the Strengthening the Reporting of Observational Studies in Epidemiology (STROBE) (Supplementary material, Table S1).

### Outcomes

Primary outcome was early clinical failure assessed 72 h after initiation of antibiotic therapy. Clinical failure was defined as the presence of one of the next criteria: the appearance of new symptoms or signs of infection, persistence or worsening of previous signs and symptoms, change in antibiotic treatment to rescue therapy and/or death.

Secondary outcomes were recurrences, unplanned readmissions and ED visits related to the SSTI in the follow-up period.

### Clinical variables and definitions

Demographic and clinical data and the Charlson comorbidity severity index were collected [15]. Previous episodes of SSTIs and history of recurrent SSTIs were also recorded. Baseline disease severity in acute infection was estimated by the following scores: the Sequential Organ Failure Assessment (SOFA) [16]; Quick SOFA score (qSOFA) [16]; Simplified Acute Physiology Score (SAPS II) [17] and the Pitt index [17] in case of bacteremia.

For the classification of SSTIs, cellulitis or erysipelas were defined as diffuse skin infection characterized by spreading areas of erythema, edema and/or induration; surgical or trauma-related wound infection was defined as infection with purulent drainage or the presence of one or more fluid collections associated with a surgical or post-traumatic wound with surrounding erythema, edema and/or induration; skin abscess was defined as an infection characterized by a collection of pus within the dermis or deeper, accompanied by erythema, edema, and/or induration, and necrotizing fasciitis was defined as a rapidly spreading skin and soft tissue infection, with progressive destruction and necrosis of the fascia and fat, with significant associated systemic toxicity [18, 19].

Reasons for admission to the hospital via the ED were classified based on electronic medical records, and comprehensive firsthand data were collected for each patient by trained investigator. Reasons were classified as: complicated infection or suspected complicated infection (deep soft tissue involvement in a patient with underlying pathology requiring intravenous antibiotic therapy and/or surgery), sepsis [20], social conditions, chronic diseases decompensation, high Charlson score or advanced age, oral intake unavailable, colonization or risk factors for multidrug-resistant infection, nonadherent patient, immunosuppression and lack of improvement after 48–72 h of treatment.

Risk factors for multidrug-resistant infection were considered in patients with previously prolonged hospitalization and/or antimicrobial exposure, invasive procedures and prior colonization or infection [21]. Immunosuppression was defined by corticosteroid use, receipt of chemotherapy or radiation therapy, diagnosis of human immunodeficiency virus or acquired immunodeficiency syndrome, absolute neutrophil count of less than 1,500/mcL, or prior solid organ or bone marrow transplantation with receipt of chronic immunosuppression [22].

Empirical antibiotic treatment was defined as antibiotics administered in the first hours of infection if cultures were not available. Definitive antibiotic treatment was defined as antibiotics administered according to the results of the antimicrobial susceptibility testing if cultures were available. Appropriate treatment was defined as antibiotic therapy administered if cultures were available and at least one of the antibiotics administered had in vitro activity against the isolated strain, according to the breakpoints established by the European Committee on Antimicrobial Susceptibility Testing (EUCAST) [23]. Antibiotic rescue therapy was defined as escalation or new empirical rescue therapy, evaluated between 24 and 72 h after the antibiotic therapy was initiated. Antibiotic adverse effects (i.e., nephrotoxicity, *Clostridioides difficile* infection, allergic reaction) were also recorded. Adequate source control, if required, was defined as percutaneous drainage or surgical intervention, as appropriate.

Clinical cure was defined if the following criteria within 72 h of initiation of antibiotic therapy were met: the patient remained afebrile (temperature less than 37.3 °C) for at least 24 h, no increase in lesion size compared to the ED consultation, decrease in the extent and intensity of one or more signs of inflammation at the primary site of infection and white blood cell (WBC) count was between 4 × 10^9/L and 12 × 10^9/L. This last criterion was added to the clinical data based on previous references [24–28]. An indeterminate clinical response was established in patients lost to follow-up. Recurrence was defined as relapse of symptoms after initial improvement of the first episode during the follow-up period. Length of hospital stay and complications during the 30 days after the onset of infection were also recorded.

Finally, in hospitalized patients a subgroup analysis was made and eligibility for early discharge (at 72 h from admission) was evaluated. The patients were eligible for early discharge if the following criteria were met: clinical cure within 72 h, absence of sepsis, septic shock and/or multiple organ failure, absence of comorbidity decompensation, no changes in cognitive or mental status or any other situation that would compromise adherence to medication at home.

### Microbiological studies

Bacterial identification was performed by conventional biochemical tests and matrix-assisted laser desorption and ionization time-of-flight mass spectrometry (MALDI-TOF MS), using the Bruker Microflex^®^ LT instrument and MALDI Biotyper^®^ software (Bruker Daltonics, MA, USA). Antibiotic susceptibility testing (AST) of isolates was performed by broth microdilution, using MicroScan panels [Beckman-Coulter] on the automated MicroScan WalkAway system [Beckman-Coulter]. Results were interpreted according to EUCAST guidelines [23].

### Statistical analysis

The required sample size was estimated at 160 patients per group and a maximum difference of 10% was considered clinically relevant. An alpha error of 0.05 and a beta error of 0.20 were set. It was calculated using a non-superiority test based on the hypothesis that the percentage of readmissions in the outpatient group would not be higher than in the hospitalized group. The results of a previous study [29] found a readmission rate of 21% in the outpatient group and 24% in the inpatient group.

Categorical variables were presented as numbers and percentages and were compared using the X^2^ test or Fisher’s exact test. Continuous variables were expressed as median and interquartile range (IQR) and compared using Student’s t-test or the Mann–Whitney U test, as appropriate.

Univariate and multivariate logistic regression analyses were used to assess associations between covariates and primary and secondary outcomes. Variables associated significantly or nearly significantly in the univariate analysis, and those that were clinically relevant but not statistically significant were included in the model. No missing imputation was applied. To avoid collinearity between related variables (homelessness and social/economic barriers), only one was included in the multivariate analysis. Results were expressed as odds ratios (OR) and their 95% confidence intervals (95% CI). Statistical analyses were performed using SPSS Statistics 26.0 software, R 4.0.0, and STATA 15.1.

### Ethics

This study was conducted in accordance with national and international guidelines of the Deontological Codel, the Declaration of Helsinki (Fortaleza, Brazil, October 2013), the Code of Good Research Practices, and Royal Decree 957/2020 of November 3rd. The study was approved by the Clinical Research Ethics Committee of the Parc de Salut Mar, Barcelona (register no. 2021/10132). Due to the observational nature of the study and retrospective analysis, the need for written informed consent was waived.

## Results

### Demographics and clinical characteristics

**Fig. 1:**
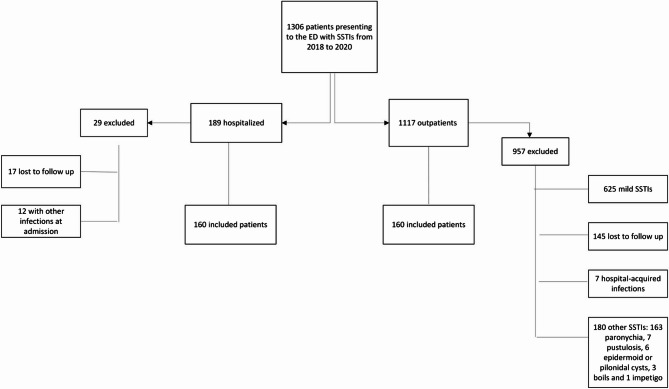
Flowchart of patients included in the study. Abbreviation: SSTIs: Skin and soft tissue infections

One thousand three hundred six patients attending the ED with SSTIs during the study period were screened. However, the vast majority of the patients did not met inclusion criteria for our study and were not included. The main reason for exclusion was the presence of mild infections that did not meet the study’s inclusion criteria. Finally, of the 1.306 patients, 320 fulfilled the inclusion and exclusion criteria and were included, of whom 50% (160 patients) were admitted in the hospital (Fig. 1). Nineteen patients (11.9%) were admitted to OPAT. Table 1 shows the differences between the groups.

**Table 1:** Baseline characteristics of patients included in the study and comparative analysis according to management

	Overall cohort (*n*=320)		
	Hospitalization (*n*=160)	Outpatients (*n*=160)	*p*-value
Demographics and social conditions			
Age (years), m (IQR)	62 (46–76)	55 (40.25–71.25)	**0.035**
Male sex	95 (59.4)	105 (65.6)	0.248
Drug injection history	19 (11.9)	13 (8.1)	0.264
Homelessness	26 (16.3)	13 (8.1)	**0.026**
Social and economic barriers to care	31 (19.4)	12 (7.5)	**0.002**
Underlying condition			
Charlson Comorbidity Index, m (IQR)	3 (1–5)	2 (0–4)	0.110
Diabetes mellitus	41 (25.6)	32 (20)	0.231
COPD	7 (4.4)	10 (6.3)	0.455
Congestive heart failure	11 (6.9)	12 (7.5)	0.829
Cirrhosis	10 (6.3)	4 (2.5)	0.101
Neurological disorder	14 (8.8)	11 (6.9)	0.532
Chronic kidney disease	23 (14.4)	11 (6.9)	**0.029**
Dialysis	3 (1.9)	4 (2.5)	1.000
Hematologic malignancy	1 (0.6)	4 (2.5)	0.371
Solid tumor malignancy	6 (3.8)	13 (8.1)	0.098
Mental illness	11 (6.9)	6 (3.8)	0.213
Immunosuppression	27 (16.9)	24 (15)	0.647
Neutropenia	0 (0)	1 (0.6)	1.000
HIV/AIDS	10 (6.3)	12 (7.5)	0.659
Kidney transplant	6 (3.8)	1 (0.6)	0.121
SSTI classification			
Cellulitis/Erysipelas	126 (78.8)	132 (82.5)	0.479
Surgical or traumatic wound infection	0 (0)	6 (3.7)	**0.030**
Skin abscess	18 (11.2)	16 (10)	0.856
Necrotizing fasciitis	2 (1.3)	0 (0.0)	0.498
Cellulitis + abscess	14 (8.8)	6 (3.8)	0.106
Localization			
Upper extremities	12 (7.5)	20 (12.5)	0.192
Lower extremities	133 (83.1)	116 (72.5)	**0.031**
Other	10 (6.25)	23 (14.4)	**0.027**
Various localizations	5 (3.12)	1 (0.62)	0.214
Portal of entry			
Surgical or traumatic wound	43 (26.9)	45 (28.1)	0.900
Ulcer	36 (22.5)	12 (7.5)	**<0.001**
Fungal infection	20 (12.5)	3 (1.9)	**0.001**
Skin lesion	8 (5)	11 (6.9)	0.636
Other	10 (6.2)	16 (10)	0.306
Unknown	43 (26.9)	73 (45.6)	**0.001**
Predisposing factors			
Lymphedema	6 (3.8)	4 (2.5)	0.748
Venous insufficiency	13 (8.1)	15 (9.4)	0.843
Obesity	3 (1.9)	6 (3.8)	0.502
Trauma	6 (3.8)	10 (6.3)	0.623
Skin disease	7 (4.4)	5 (3.1)	0.556
Fungal infection	16 (10)	3 (1.9)	**0.002**
Diabetic foot	4 (2.5)	1 (0.6)	0.371
Stasis dermatitis	1 (0.6)	2 (1.3)	1.000
Drug injection-related wound	12 (7.5)	9 (5.6)	0.498
Others	22 (13.8)	13 (8.1)	0.152
None	46 (28.7)	84 (52.5)	**<0.001**
Various factors	28 (17.5)	3 (1.9)	**<0.001**
HCA Risk Factors			
Hospitalization in last 3 months	23 (14.4)	16 (10)	0.232
Surgery in last 3 months	3 (1.9)	10 (6.3)	**0.047**
Residence in a long-term care facility	6 (3.8)	0 (0)	**0.030**
Antibiotic exposure in last 3 months	59 (36.9)	47 (29.4)	0.154
Admission to long-term care facility	3 (1.9)	0 (0)	0.248
*P*revious episodes of SSTIs	53 (33.1)	22 (13.8)	**<0.001**
*Recurrent* SSTIs	44 (27.5)	13 (8.1)	**<0.001**
Laboratory parameters on ED admission			
CRP,m (IQR) (mg/dL)	11.5 (5.3–22.36)	5.1 (2.4–10.6)	**<0.001**
WBC,m (IQR) (x10E3/µL)	14.3 (9.3–18.9)	10.7 (7.7–13.1)	**<0.001**
Positive culture	64 (40)	18 (11.3)	**<0.001**
Blood culture	11 (16.9)	2 (11.1)	0.724
Drainage culture	21 (32.3)	11 (61.1)	0.051
Wound smear culture	30 (46.2)	5 (27.8)	0.260
Various cultures	3 (4.6)	0 (0)	1.000
Baseline Disease Severity			
SOFA score, m (IQR)	0 (0–1)	0 (0-0)	**0.018**
qSOFA score, m (IQR)	0 (0-0)	0 (0-0)	**0.002**
SAPS II, m (IQR)	26 (19–31.75.75)	19 (19–28)	**<0.001**
Sepsis or septic shock	11 (6.9)	0 (0)	**0.002**
ICU admission	5 (3.1)	0 (0)	0.061
Bacteraemia	12 (7.5)	2 (1.3)	**0.006**
Pitt score, m (IQR)	0 (0-0)	0 (0-0)	0.220
Management			
Appropriate treatment	58 (90.6)	12 (66.6)	**<0.001**
Inappropriate treatment	3 (4.6)	7 (38.8)	0.218
72 h delay in initiating appropriate antibiotic therapy	5 (7.8)	0 (0)	0.576
Source control			
Surgery	26 (16.5)	1 (0.6)	**<0.001**
Percutaneous drainage	7 (4.4)	16 (10)	0.055
Not required	125 (79.1)	143 (89.4)	**0.012**
Inadequate	3 (1.9)	0 (0)	0.248
Discharge against medical advice	16 (10)	7 (4.4)	0.051
Outpatient treatment			
Oral antibiotic	76 (47.5)	157 (98.1)	**<0.001**
Day-care hospital	1 (0.6)	0 (0)	1.000
“Hospital at home” program	3 (1.9)	0 (0)	0.248
Not completed	2 (2.5)	7 (4.5)	0.722
Completed	57 (71.3)	134 (85.4)	**0.009**
Unknown	21 (26.3)	16 (10.2)	**0.001**
Antibiotic Rescue Therapy			
Yes	12 (7.5)	21 (13.1)	0.098
No	144 (90)	139 (86.9)	0.382
Unknown	4 (2.5)	0 (0)	0.123
Antibiotic-related side effects	16 (10)	8 (5)	0.093
Acute kidney injury	2 (12.5)	0 (0)	0.520
* Clostridioides difficile*	3 (18.8)	0 (0)	0.280
Diarrhea negative for* Clostridioides difficile*	4 (25)	3 (33.3)	0.673
Allergic reaction	4 (25)	5 (55.6)	0.200
Other	3 (18.8)	1 (11.1)	1.000

Data are presented as nos. (%) unless otherwise specified

*Abbreviations*: *AIDS* acquired immunodeficiency syndrome, *COPD* chronic obstructive pulmonary disease, *CRP* C-reactive protein, *ED* emergency department, *HCA* healthcare-acquired, *HIV* human immunodeficiency virus, *ICU* intensive care unit, *IQR* interquartile range, *m* median, *SAPS II* Simplified Acute Physiology Score, *SOFA* Sequential Organ Failure Assessment, *SSTI(s)* skin and soft tissue infection(s), *WBC* white blood cell count

In the hospitalized group, the most common reasons for admission to hospital were (patients could have more than one reason for admission): complicated infection or suspected complicated infection (31/160, 19.3%), colonization or risk of infection with multidrug-resistant microorganisms (27/160, 16.8%), consultation to the ED due to lack of improvement after 48–72 h of treatment (27/160, 16.8%), chronic diseases decompensation (23/160 14.3%), risk of non-adherence to treatment (15/160, 9.3%), social and economic barriers to regular care (15/160, 9.3%), sepsis or septic shock (11/160, 6.8%), immunosuppression (7/160, 4.3%) and advanced age/high Charlson score (12/160, 7.5%).

In hospitalized patients, 31 had complications related with the admission. The most frequent complications were phlebitis (9/160, 5.6%), delirium (8/160, 5%) and nosocomial infections (7/160, 4.3%). In the OPAT cohort only one patient with phlebitis had a health-care related complication during admission.

The median length of hospital stay was 9 days (IQR 5–15), and 20/160 patients (12.5%) were discharged within the first 72 h. Ninety seven out of 160 (60.6%) hospitalized patients were not eligible for early discharge. The most common reasons for ineligibility for early discharge were (patients could have more than one reason): clinical cure criteria not met (25/97, 25.7%), need for parenteral antibiotic therapy or wound care (21/97, 21.6%), social and economic barriers to regular care (13/97, 13.4%), sepsis/septic shock (11/97 11.3%), chronic diseases decompensation (10/97, 10.3%), cognitive or mental disorders (7/97, 7.2%) and alcohol or substance use disorder (3/97, 3%). Parenteral antibiotic therapy was required for one or more of the following reasons: suspicion of infection with a multidrug-resistant organism (based on colonization, positive culture results, known risk factors or prior treatment failure), for which effective treatment options were limited to the parenteral route; and the patient’s inability to tolerate oral intake. Twenty-three patients (7%) left against medical advice; 16/23 (69.5%) of this group were PWID and/or homeless.

### Baseline illness severity, microbiological information and therapeutic management

At the time of first assessment, hospitalized patients were more seriously ill, as indicated by worse laboratory parameters (CRP and WBC, *p* < 0.001 in both cases), higher severity scores (SOFA (*p* = 0.018), qSOFA (*p* = 0.002) and SAPS II (*p* < 0.001)) and a higher proportion of sepsis or septic shock (*p* < 0.001). Among inpatients, 11 patients (6.9%) had sepsis or septic shock but only 5 patients (3.1%) required admission to the ICU.

**Fig. 2:**
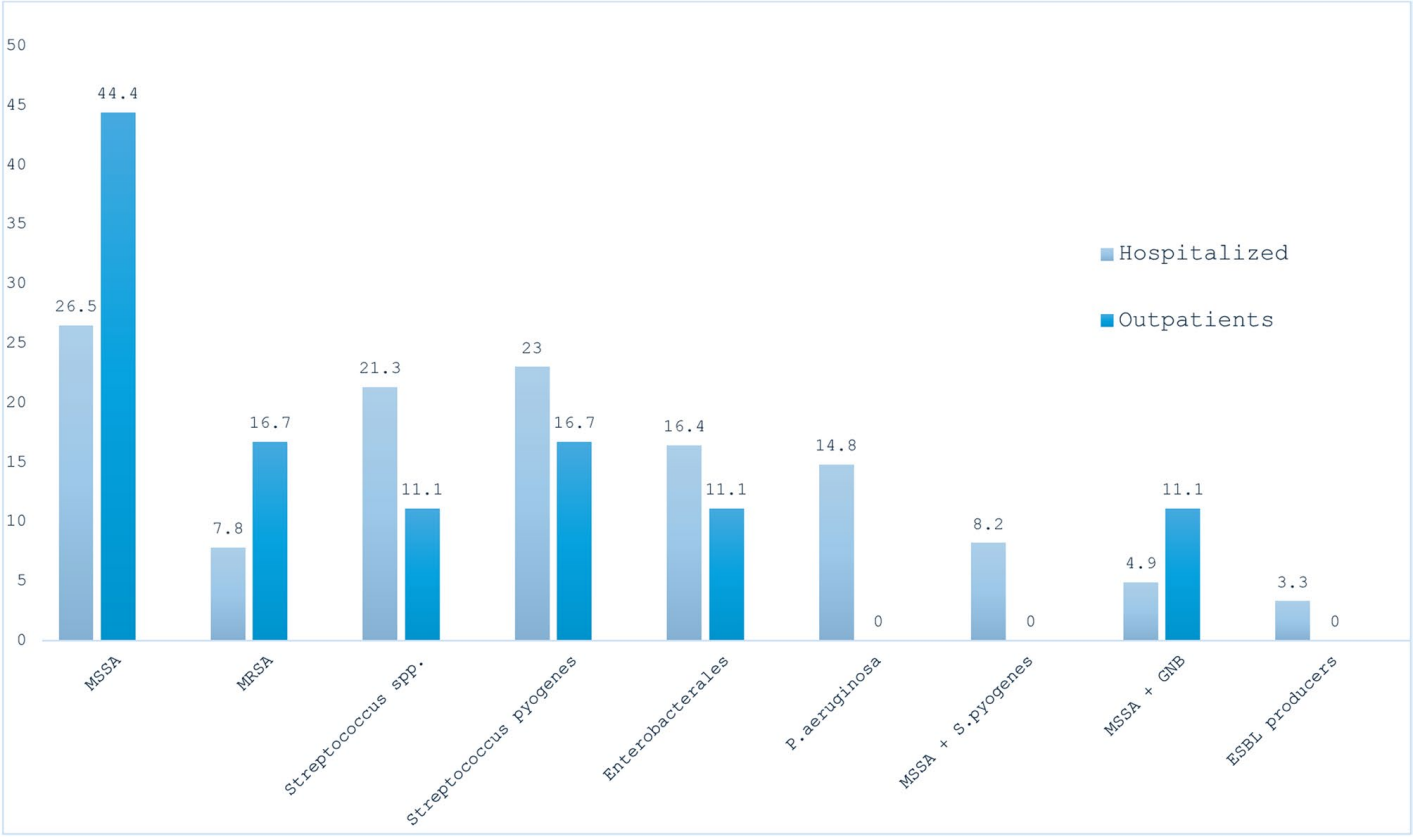
Microbiological data by study group (hospitalized vs. outpatients). Abbreviations: GNB (gram negative bacilli), ESBL (extended- spectrum beta-lactamases), MRSA (methicillin-resistant *Staphylococcus aureus*), MSSA (methicillin-susceptible *Staphylococcus aureus*), P. aeruginosa (*Pseudomonas aeruginosa*). All MRSA isolates were susceptible to clindamycin and trimethoprim/sulfamethoxazole, and 3/8 isolates (37.5%) were susceptible to levofloxacin. For vancomycin, half the strains (4/8) had a minimum inhibitory concentration (MIC) = 1 µg/ml and the other half had a MIC = 2 µg/ml. Among Enterobacterales, 10/15 (66%) were resistant to amoxicillin/clavulànic acid, 5/15 (33.3%) were resistant to ciprofloxacin. In *Pseudomonas aeruginosa* infections, 4/9 (44.4%) were resistant to ciprofloxacin

Among patients with microbiological results (82/320, 25.6%), *Staphylococcus aureus* was the most common pathogen isolated in both groups. Figure 2 shows the microbiological data by study group.

The most common empiric antimicrobial therapies used in hospitalized patients were amoxicillin/clavulanic acid (75/160, 46.9%), ciprofloxacin plus clindamycin (23/160, 14.4%), piperacillin/tazobactam (21/160, 13.1%), piperacillin/tazobactam plus vancomycin (17/160, 10.6%) and ceftriaxone plus clindamycin (10/160, 6.3%). Among admitted patients with microbiological isolation (*n* = 64), 90.6% received appropriate antibiotic treatment. In the outpatient setting, the most common antibiotics used were oral amoxicillin/clavulanic acid (116/160, 73.9%) and oral ciprofloxacin plus clindamycin (28/160, 17.8%). Two patients were managed with long-acting lipoglycopeptides (both dalbavancin as outpatients).

### Primary objective: clinical outcomes

#### Early clinical failure

Clinical failure assessed 72 h after initiation of antibiotic therapy was 20.7% (31/150) and 17.3% (26/150) in the outpatient and inpatient groups, respectively (*p* = 0.467). Among patients admitted to OPAT, 2/19 (10.5%) had early clinical failure.

The unadjusted and adjusted analysis of variables associated with clinical failure are shown in Table 2. After adjusting for confounders in multivariate analysis, there were no differences according to management in early clinical failure. Clinical failure was independently associated with diabetes mellitus (OR 2.75, 95% CI 1.29–5.87; *p* = 0.009).

**Table 2:** Univariate and multivariate analysis of parameters predicting early clinical failure

	Early clinical failure cohort sample (*n*= 300)						
	Clinical Failure (*n* =57)	Non-Clinical Failure (*n* =243)	Unadjusted OR (95% CI)	*p-value*		Adjusted OR (95% CI)	*p-value*
Management							
Outpatient	31 (20.7)	119 (79.3)	-		-	1.00	
Hospitalization	26 (17.3)	124 (82.7)	0.80 (0.45–1.44.45.44)		0.467	0.68 (0.37–1.26)	0.223
Demographics and social conditions							
Age (years), m (IQR)	59 (44–76)	59 (44–74)	1.00 (0.98–1.02)		0.970	0.99 (0.97–1.02)	0.606
Male sex	39 (21.4)	143 (78.6)	-		-	1.00	
Female sex	18 (15.3)	100 (84.7)	0.66 (0.36–1.22)		0.186	0.71 (0.37–1.37)	0.311
History of drug injection	9 (32.1)	19 (67.9)	2.21 (0.94–5.18)		0.082	1.70 (0.59–4.91)	0.324
Homelessness	11 (34.4)	21 (65.6)	2.53 (1.14–5.60)		**0.030**	2.66 (0.99-7.10)	0.052
Social and economic barriers to care	10 (27.8)	26 (72.2)	1.78 (0.80–3.93)		0.170		
Underlying conditions							
Charlson Comorbidity Index, m(IQR)	2.00 (0–6)	2 (0–4)	1.06 (0.96–1.16)		0.266	1.07 (0.91–1.26)	0.433
Diabetes mellitus	21 (30)	49 (70)	2.31 (1.24–4.31)		**0.011**	2.75 (1.29–5.87)	**0.009**
COPD	6 (37.5)	10 (62.5)	2.74 (0.95–7.88)		0.078		
Congestive heart failure	1 (4.55)	21 (95.5)	0.19 (0.02–1.43)		0.061		
Cirrhosis	1 (7.69)	12 (92.3)	0.34 (0.04–2.70)		0.316		
Neurological disorder	5 (20.8)	19 (79.2)	1.13 (0.40–3.18)		0.787		
Chronic kidney disease	9 (27.3)	24 (72.7)	1.71 (0.75–3.91)		0.216		
Hematologic malignancy	2 (40)	3 (60)	2.91 (0.47–17.8)		0.291		
Solid tumor malignancy	3 (15.8)	16 (84.2)	0.79 (0.22–2.80)		0.759		
Mental illness	5 (35.7)	9 (64.3)	2.50 (0.80–7.77)		0.136		
Immunosuppression	11 (25)	33 (75)	1.52 (0.72–3.23.72.23)		0.283		
HIV/AIDS	1 (6.67)	14 (93.3)	0.29 (0.04–2.27)		0.223		
Neutropenia	0 (0)	1 (100)	0.00 (0.00;.)		0.810		
Kidney transplant	1 (14.3)	6 (85.7)	0.71 (0.08–5.98)		0.829		
SSTI classification							
Cellulitis/Erysipelas	44 (18.2)	198 (81.8)	-		-		
Surgical or traumatic wound infection	2 (33.3)	4 (66.7)	2.25 (0.40–12.7.40.7)		0.388		
Skin abscess	11 (22)	39 (78)	1.27 (0.60 −2.67)		0.527		
Necrotizing fasciitis	0 (0)	2 (100)	0.00 (0.00;.)		0.671		
Localization							
Lower extremities	44 (18.9)	189 (81.1)	-		-		
Upper extremities	4 (13.8)	25 (86.2)	0.69 (0.23–2.08)		0.533		
Other	7 (21.9)	25 (78.1)	1.20 (0.49–2.96)		0.675		
Various localizations	2 (33.3)	4 (66.7)	2.15 (0.38–12.1)		0.413		
Portal of entry							
Surgical or traumatic wound	20 (25)	60 (75)	-		-		
Ulcer	11 (22.9)	37 (77.1)	0.89 (0.38–2.07)		0.801		
Fungal infection	1 (4.35)	22 (95.7)	0.14 (0.02–1.08)		**0.025**		
Skin lesion	3 (15.8)	16 (84.2)	0.56 (0.15-2.13)		0.420		
Others	6 (23.1)	20 (76.9)	0.90 (0.32–2.55)		0.865		
Unknown	16 (15.4)	88 (84.6)	0.55 (0.26–1.14)		0.110		
Predisposing factors							
None	21 (17.2)	101 (82.8)	-		-		
One or more factors	36 (20.2)	142 (79.8)	1.22 (0.67–2.21)		0.521		
*P*revious episodes of SSTIs	13 (18.3)	58 (81.7)	0.94 (0.47- 1.87)		0.881		
*Recurrent *SSTIs	10 (19.2)	42 (80.8)	1.02 (0.48–2.18)		0.944		
Hospital-at-home	2 (10.5)	17 (89.5)	0.48 (0.11–2.15)		0.355		
Voluntary discharge	7 (53.8)	6 (46.2)	5.53 (1.78–17.2)		**0.005**		
Baseline illness severity							
SOFA score, m (IQR)	0 (0–1)	0 (0–1)	1.00 (0.80–1.26)		0.979		
SAPS II	24 (19–30)	24 (19–30)	1.01 (0.98–1.04)		0.670		
Sepsis	1 (25)	3 (75)	1.42 (0.14–13.9)		0.740		
Septic shock	1 (14.3)	6 (85.7)	0.71 (0.08–6.01.0)		0.833		
ICU admission	1 (20)	4 (80)	1.07 (0.12–9.73)		0.896		
Bacteremia	5 (35.7)	9 (64.3)	2.50 (0.80–7.77)		0.136		
Therapeutic management							
Appropriate treatment	15 (22.1)	53 (77.9)	1.31 (0.67–2.55)		0.426		
72 h delay in initiating appropriate antibiotic therapy	2 (40)	3 (60)	2.56 (0.39–17)		0.371		
Source control							
Not required	47 (18.7)	205 (81.3)	-		-		
Surgery	8 (32)	17 (68)	2.05 (0.84–5.04)	0.132	0.132		
Percutaneous drainage	1 (4.55)	21 (95.5)	0.21 (0.03.1.58)	0.085	0.085		

Data are presented as nos. (%) unless otherwise specified

*Abbreviations*: *AIDS* acquired immunodeficiency syndrome, *COPD* chronic obstructive pulmonary disease, *ED* emergency department, *HIV* human immunodeficiency virus, *ICU* intensive care unit, *IQR* interquartile range, *m* median, *SAPS II* Simplified Acute Physiology Score, *SOFA* Sequential Organ Failure Assessment, *SSTI(s)* skin and soft tissue infection(s)

#### Secondary outcomes: recurrences, unplanned readmissions and ED visits related to the SSTI

Recurrence was 7.5% (12/160) and 12.3% (19/155) in the outpatient and inpatient groups, respectively (*p* = 0.163). Among patients admitted to OPAT, recurrence was 5.26% (1/19). Unplanned readmissions related to the SSTI were 16.2% (26/160) and 17% (27/159) in the outpatient and inpatient groups, respectively (*p* = 0.862). Among patients admitted to OPAT, 15.8% were readmited (3/19). ED visits related to the SSTI were 25.6% (41/160) and 17.5% (28/160) in the outpatient and inpatient groups, respectively (*p* = 0.079). Among patients admitted to OPAT, 15.8% (3/19) visited the ED in the follow-up period (3/49).

The unadjusted and adjusted analysis of variables associated with recurrences, unplanned readmissions and ED visits related to the SSTI are shown in Suplementary Tables S2, S3 and S4, respectively. After adjusting for confounders in multivariate analysis, there were no differences according to management in recurrence and unplanned readmissions.

Recurrence was independently associated with history of recurrent SSTIs (OR 5.86, 95% CI 2.51–13.67; *p* < 0.001). Unplanned readmissions were indepently associated with diabetes mellitus (OR 2.04, 95% CI 1.01–4.10; *p* = 0.046) and SOFA score (OR 1.25, 95% CI1.01–1.53; *p* = 0.039).

Hospitalization was independently associated with ED visits related to the SSTI with inpatients showing significantly lower odds of ED revisits (OR 0.32, 95% CI 0.16–0.62; *p* = 0.001). Moreover, ED visits related to the SSTI were indepently asociated with homelesness (OR 3.35, 95% CI 1.32–8.46; *p* = 0.011) and history of recurrent SSTIs (OR 6.47, 95% CI 3.05–13.71; *p* < 0.001).

### Secondary objective: independent factors associated with eligibility for early discharge in hospitalized patients

Sixty-three out of 160 (39.3%) hospitalized patients were eligible for early discharge. Crude and adjusted analyses of variables associated with meeting the eligibility criteria for early discharge are shown in Table 3. In the multivariate analysis, immunosuppression was independently associated with fulfilling the criteria for eligibility for early discharge (OR 3.02 95% CI 1.13–8.10; *p* = 0.028). SAPS II score (OR 0.92 95% CI 0.88–0.96; *p* < 0.001) and social and economic barriers to regular care (OR 0.19 95% CI 0.07–0.54; *p* = 0.002) were associated with not fulfilling the criteria for eligibility for early discharge.

**Table 3:** Univariate and multivariate analysis of parameters predicting fulfillment of eligibility criteria for early discharge in hospitalized patients

	Total cohort (*n* = 160), met eligibility criteria for early discharge =83)						
	Criteria met (*n* =83)	Criteria not met (*n* =77)	Unadjusted (95% CI)	OR	*p-value*	Adjusted OR (95% CI)	*p-value*
Demographics and social conditions							
Age (years), m (IQR)	63 (48–71)	64 (46–79.50)	1.02	(1.00–1.04)	**0.033**		
Female sex	34 (41)	31 (40.2)	0.97	(0.52–1.83)	0.929	0.92 (0.45–1.90)	0.827
History of drug injection	6 (7.2)	13 (16.9)	2.61	(0.94–7.25)	0.066		
Homelessness	8 (9.6)	18 (23.4)	2.86	(1.16–7.03)	**0.021**		
Social and economic barriers to care	9 (10.8)	22 (28.6)	3.29	(1.41–7.70)	**0.005**	0.19 (0.07-0.54)	**0.002**
Underlying conditions							
Charlson Comorbidity Index, m(IQR)	3 (1–4)	3 (1–6)	1.15	(1.02–1.29)	**0.024**		
Diabetes mellitus	17 (20.5)	24 (31.1)	1.76	(0.86–3.61)	0.128		
COPD	2 (2.4)	5 (6.5)	2.81	(0.53–14.9)	0.239		
Congestive heart failure	5 (6)	6 (7.8)	1.32	(0.39–4.51)	0.673		
Cirrhosis	3 (3.6)	7 (9)	2.67	(0.66–10.7)	0.173		
Neurological disorder	7 (8.4)	7 (9)	1.09	(0.36–3.25)	0.886		
Chronic kidney disease	10 (12)	13 (16.9)	1.48	(0.61–3.61)	0.396		
Dialysis	2 (2.4)	1 (1.3)	0.53	(0.05–6.00)	0.665		
Hematologic malignancy	0 (0)	1 (1.3)	-		0.481		
Solid tumor malignancy	2 (2.4)	4 (5.2)	2.22	(0.39–12.5)	0.394		
Mental illness	4 (4.8)	7 (9)	1.98	(0.55–7.03)	0.309		
Immunosuppression	19 (22.9)	8 (10.3)	0.39	(0.160-0.95)	**0.037**	3.02 (1.13–8.10)	**0.028**
HIV/AIDS	6 (7.2)	4 (5.2)	0.70	(0.19–2.59)	0.618		
Kidney transplant	3 (3.6)	3 (3.9)	1.08	(0.21–5.52)	0.929		
HCA Risk Factors							
Hospitalization in last 3 months	14 (16.8)	9 (11.7)	0.65	(0.26–1.61)	0.363		
Surgery in last 3 months	1 (1.2)	2 (2.6)	2.19	(0.19–24.6)	0.581		
Antibiotic exposure in last 3 months	38 (45.7)	21 (27.3)	0.44	(0.23–0.86)	**0.016**	1.69 (0.80–3.58)	0.169
Previous episodes of SSTIs	30 (36.1)	23 (29.9)	0.75	(0.39–1.46)	0.406		
Recurrent SSTIs	24 (29)	20 (26)	0.86	(0.43–1.73)	0.683		
Laboratory parameters on ED admission							
CRP, m (IQR) (mg/dL)	11.8 (3.5–23.1)	14.9 (6.3–27.8)	1.00	(0.99–1.01)	0.874		
Leukocytes, m (IQR) (x10E3/µL)	13.6 (10.5–18.4)	14.9 (9.2–19.7)	1.03	(0.98–1.08.9)	0.249		
Positive culture	28 (33.7)	36 (46.7)	1.72	(0.91–3.27)	0.097		
Blood culture	3 (10.3)	8 (22.2)	2.42	(0.50–11.8)	0.299		
Baseline illness severity							
SOFA score, m (IQR)	0 (0-0)	1 (0–2)	1.72	(1.24–2.39)	**0.001**		
SAPS II	24 (19–30)	29 (19–36)	1.07	(1.03–1.11)	**0.001**	0.92 (0.88–0.96.)	**<0.001 **
Management							
Appropriate treatment	25 (43.1)	33 (56.9)	0.66	(0.06–7.69)	0.794		
Source control	16 (48.5)	17 (51.5)	1.15	(0.53–2.48)	0.724		
S﻿urgery	12 (46.2)	14 (53.8)	1.26	(0.54–2.95)	0.596		
Percutaneous drainage	4 (57.1)	3 (42.9)	0.81	(0.17–3.78)	0.810		
Antibiotic Rescue Therapy	2 (16.7)	10 (83.3)	5.75	(1.22–27.2)	**0.016**		
Antibiotic-related side effects	4 (25)	12 (75)	3.65	(1.12–11.8)	**0.027**		

Data are presented as nos. (%), unless otherwise specified

*Abbreviations*: *AIDS* acquired immunodeficiency syndrome, *COPD* chronic obstructive pulmonary disease, *CRP* C-reactive protein, *ED* emergency department, *HCA* healthcare-acquired, *HIV* human immunodeficiency virus, *IQR* interquartile range, *ICU* intensive care unit, *m* median, *SAPS II* Simplified Acute Physiology Score, *SOFA* Sequential Organ Failure Assessment, *SSTIs* skin and soft tissue infections

## Discussion

In the present study comparing the management of community-acquired SSTIs, we found no differences in early clinical failure, recurrence and unplanned readmissions between inpatients and outpatients. Numerous studies have set out to analyse the clinical characteristics and various outcomes in SSTIs, but to our knowledge, few have compared the differences between inpatient and outpatient approaches. After reviewing the literature, we found only one previous study, by Bookstaver et al., that compared the two groups [29]. They found that hospitalized patients were older and had more comorbidities than outpatients. No differences in unplanned ED visits or readmissions were observed between the groups. Consistent with these results, no differences were observed in the present study in either early clinical cure, recurrences, and readmissions related to the SSTI. Alternatively, in the current study patients who were hospitalized had significantly reduced odds of returning to the ED for SSTI-related concerns, suggesting a protective effect of hospitalization. This finding may be related with the higher rate of appropriate treatment in inpatients. On the contrary, some outpatients may return to the ED early after starting oral antibiotics, before sufficient time has passed for clinical improvement, as defervescence can be slower. This early reconsultation may reflect caution rather than true treatment failure.

In the present cohort, hospitalized patients had higher severity scores at the onset of infection, as assessed by SOFA, qSOFA and SAPS II scores. In addition, infection severity evaluated by SAPS II score was associated with not meeting the eligibility criteria for early discharge and severity evaluated by SOFA score was associated with unplanned readmissions related to the SSTIs. Despite the high frequency of SSTIs in the community, there is no validated score to assess the severity of infection and guide decisions about where patients should receive care. Several severity scoring systems have been developed but none has been validated in prospective multicentre cohort studies or clinical trials [30]. Furthermore, although 39% of hospitalized patients fulfilled predefined criteria for early discharge, only 12.5% were actually discharged within the first 72 h. This discrepancy reflects the gap between clinical eligibility and real-world practice. Several contributing factors have been described, including institutional and structural barriers, as well as logistical and social challenges [31].

In the present and other series, homeless patients and patients with economic/social barriers to regular care, such as those with a history of injecting drug use, represent a vulnerable population with a dramatic increase in the incidence of SSTIs and hospitalization in recent years [32]. Many of these patients use strategies to delay or avoid seeking medical attention due to fear of stigma and discrimination from healthcare professionals, as well as structural barriers to accessing medical care [33]. As a result, these patients have increased disease severity, longer courses of antibiotics and hospitalizations, high readmission rates and lack of appropriate follow-up [34]. In this study, patients with social and economic barriers to regular care were associated with a lower likelihood of being eligible for early discharge and homeless patients were associated with a higher probabilty of returning to the ED attributable to the SSTIs. In addition, 11/32 (34%) of those classified as PWID were discharged against medical advice, which is slightly higher than the rates of up to 30% reported in previous studies [34, 35].

Retrospective reports of newer long-acting antibiotics have shown the effectiveness and safety of these agents in the general population [36] and similar or even better results in terms of efficacy, safety and reduction in length of hospital stay compared to standard-of-care in patients with social and economic barriers to care and PWID [37]. Therefore, long-acting glycopeptides could be a useful treatment option in patients with low adherence, but further multicentre prospective studies are needed to confirm the results.

SSTIs are among the most common infections treated in OPAT units [38]. In a recent systematic review and meta-analysis of clinical trials comparing the efficacy and safety of OPAT versus inpatient parenteral antimicrobial therapy, no differences were found for mortality, treatment failure or adverse reactions related to antimicrobials, or the device used to administer them [8]. In the present cohort, 19 patients were enrolled in the OPAT program. Early clinical cure in this group was 89.5%, which is similar to previously published studies [39, 40]. Secondary to the SSTI 15.8% visited the ED and 15.8% of the patients were readmitted in the 30-day follow-up period. In another series, the readmission rates for patients with SSTIs ranged from 0% to 21% [39, 41–43]. In the OPAT cohort, none of the patients was PWID. Regarding the management of PWID in the outpatient setting, there is evidence that OPAT may be safe in these patients [44]. However, other studies have reported that PWID patients may not adhere to treatment and so experience readmissions and catheter-related complications such as drug relapse [45].

Finally, regarding the aetiology of SSTIs in this population, *S. aureus* and *Streptococcus* were the most common isolates among patients with microbiological data, with a rate of 40.2% (34.3% in hospitalized patients and 61% in outpatients) of *S.aureus* isolates and 39% (84.3% in hospitalized patients and 15.6% in outpatients) of *Streptoccus* isolates. Among *S. aureus* isolates, methicillin-susceptible *Staphylococcus aureus* was more prevalent than MRSA (25/33, 75.7%, vs. 8/33, 24.2%). The rates of *S.aureus in* hospitalized patients were similar to those reported in other European studies [46] and significantly lower than those reported in North America, with values ranging from 35.9% to 81% of specimens isolated by culture [46, 47]. Among outpatients, the rate of *S. aureus* in the present study was significantly higher than that reported in previous studies, with values around 39% [48]. Previous reports of MRSA in SSTIs in Spain have found rates from 13 to 24.6% of the *S. aueus* isolates [49], while in Europe the rates rise to 29% with large variations among countries (higher prevalences in the Southern countries) [46]. In Latin and North America, the rates are more worrisome, with reports of rates up to 59% in North America [46] and up to 88% in Latin America with significant regional difference [50]. For gram-negative bacteria, Enterobacterales were isolated in 17% of cultures and *Pseudomonas aeruginosa* in 14.8% of hospitalized patients, rates consistent with previous studies in Europe and North America [46].

This study has some limitations that should be acknowledged. First, those related to the retrospective design with possible reporting bias. Outpatient evolution was assessed through visits to the hospital and primary care centers by reviewing patients’ medical records through the electronic health record system of the Catalonia healthcare region. However, in some cases, early clinical response may not have been assessed precisely at the 72-hour mark. Second, the higher rate of appropriate antibiotic treatment observed in the hospitalized group may reflect the use of broader-spectrum empirical therapy in these patients, who generally present with greater severity of illness and higher comorbidity. As a result, the improved appropriateness of initial treatment in this group could have contributed to better outcomes introducing potential performance bias. Third, we highlight possible selection biases; as this was a single-centre study including an idiosyncratic population attributable to an area of influence of Barcelona with a high prevalence of patients with social and economic barriers to care and PWID. In addition, necrotizing fasciitis is an infection that always requires hospital admission, although it may initially be managed in the outpatient setting until a definitive diagnosis is established. Therefore, these results may not be generalizable to other hospitals with different populations. Fourth, the sample size calculation was not based on the primary outcome, clinical failure, but rather on the secondary outcome of readmission. This was due to the lack of published studies specifically evaluating clinical failure, which limited our ability to estimate an expected effect size. Although this approach ensured sufficient statistical power, it may reduce the precision of our findings related to the primary outcome and affect the robutness of our study. Finally, the present study was conducted between 2018 and 2020. In 2020, the SARS-CoV-2 outbreak became a global pandemic [51] and the number of SSTI visits to the ED and hospitalizations may have decreased due to the prioritization of outpatient management. In addition, the use of long-acting glycopeptides was still infrequent in our hospital during the study period. Therefore, the outpatient management performed in the present study may not accurately reflect the management performed today, with more common use of long-acting antibiotics. However, several strengths of this study can be highlighted. Although previous reports have analysed the characteristics of SSTIs in inpatients and outpatients, to our knowledge, this is one of the first studies to compare differences between groups. Finally, we analysed factors associated with suitability for potential early discharge, a strategy that can be used by health care systems to reduce the burden associated with hospital admissions.

## Conclusions

The present study found no differences in early clinical failure, recurrences and unplanned readmissions related to the SSTI between inpatients and outpatients in community-acquired SSTIs. Social and economic barriers to regular care and infection severity were identified as factors associated with unsuitability for early discharge in hospitalized patients.

These results warrant further investigation to establish standard criteria for hospital admission in SSTIs. Expanding outpatient management by using resources such as early discharge and switching to oral therapy, long-acting glycopeptides, and OPAT may represent an opportunity to reduce the healthcare burden, while improving patient adherence to care and clinical outcomes.

## Supplementary Information

Supplementary Material 1.

Supplementary Material 2.

Supplementary Material 3.

Supplementary Material 4.

## Data Availability

All data generated or analysed during this study are included in this published article [and its supplementary information files].

## Abbreviations

SSTIs: Skin and soft tissue infections
ED: Emergency department
MRSA: Methicillin-resistant *Staphylococcus aureus*
PWID: Persons who inject drugs
OPAT: Outpatient parenteral antimicrobial therapy
GPC: Gram- positive cocci
STROBE: Reporting of Observational Studies in Epidemiology
SOFA: Sequential Organ Failure Assessment
qSOFA: Quick SOFA score
SAPS II: Simplified Acute Physiology Score
EUCAST: European Committee on Antimicrobial Susceptibility Testing
WBC: White blood cell count
MALDI-TOF MS: Matrix-assisted laser desorption and ionization time-of-flight mass spectrometry
AST: Antibiotic susceptibility testing
IQR: Interquartile range
OR: Odds ratios
CI: Confidence intervals
CRP: C reactive protein
ICU: Intensive Care Unit
