# Supplementary material for: Inpatient versus outpatient management of community-acquired acute skin and soft tissue infections. Clinical outcomes and factors associated with eligibility for early discharge
Source: BMC Infect Dis. 2025 Nov 17;25:1594. doi: 10.1186/s12879-025-11883-6 (PMC12625354; doi:10.1186/s12879-025-11883-6)
Supplement: Supplementary file 1 — Supplementary Material 1. [file 12879_2025_11883_MOESM1_ESM.docx]

**Supplementary Table S1. Checklist of items according to STROBE document.**

|  | Item No | Recommendation | Assessment in article |
| --- | --- | --- | --- |
| **Title and abstract** | 1 | (*a*) Indicate the study’s design with a commonly used term in the title or the abstract | a) Study design specified in the abstract. |
|  |  | (*b*) Provide in the abstract an informative and balanced summary of what was done and what was found | b) Informative and balanced summary present in the abstract. |
| Introduction | | |  |
| **Background/****rationale** | 2 | Explain the scientific background and rationale for the investigation being reported | The scientific background and rationale are described in the Introduction. |
| **Objectives** | 3 | State specific objectives, including any prespecified hypotheses | Specific objectives and prespecified hypotheses are explained in the Introduction. |
| Methods | | |  |
| **Study design** | 4 | Present key elements of study design early in the paper | Study design is presented in the first part of Methods. |
| **Setting** | 5 | Describe the setting, locations, and relevant dates, including periods of recruitment, exposure, follow-up, and data collection | Described in Methods. |
| **Participants** | 6 | (*a*) Give the eligibility criteria, and the sources and methods of selection of participants. Describe methods of follow-up | a) Described in Methods. |
|  |  | (*b*) For matched studies, give matching criteria and number of exposed and unexposed | b) This is not a matched study. |
| **Variables** | 7 | Clearly define all outcomes, exposures, predictors, potential confounders, and effect modifiers. Give diagnostic criteria, if applicable | Present in Methods. |
| **Data sources/** **measurement** | 8* | For each variable of interest, give sources of data and details of methods of assessment (measurement). Describe comparability of assessment methods if there is more than one group | Explained in Methods. The same methods for data collection were used in both groups. |
| **Bias** | 9 | Describe any efforts to address potential sources of bias | Selection bias: Inclusion of consecutive cases.  Information bias: Use of well-defined variables that are easy to collect.  Use of soft and hard outcome variables. |
| **Study size** | 10 | Explain how the study size was arrived at | Calculation of the estimated sample size is explained in Methods. |
| **Quantitative** **variables** | 11 | Explain how quantitative variables were handled in the analyses. If applicable, describe which groupings were chosen and why | Quantitative variables were handled as such. No groupings were created. |
| **Statistical** **methods** | 12 | (*a*) Describe all statistical methods, including those used to control for confounding | a) Statistical analysis is explained in Methods. |
|  |  | (*b*) Describe any methods used to examine subgroups and interactions | b) Not applicable. |
|  |  | (*c*) Explain how missing data were addressed | c) Variables with >20% missing values were not included in multivariate analysis. Included in Methods |
|  |  | (*d*) If applicable, explain how loss to follow-up was addressed | d) Patients lost to follow-up were excluded from analysis. Explained in Methods. |
|  |  | (*e*) Describe any sensitivity analyses | Not applicable. |
| Results | | |  |
| **Participants** | 13* | (a) Report numbers of individuals at each stage of study—eg numbers potentially eligible, examined for eligibility, confirmed eligible, included in the study, completing follow-up, and analysed | Explained in Results and Figure 1. |
|  |  | (b) Give reasons for non-participation at each stage | Not applicable |
|  |  | (c) Consider use of a flow diagram | Figure 1 |
| **Descriptive** **data** | 14* | (a) Give characteristics of study participants (eg demographic, clinical, social) and information on exposures and potential confounders | Table 1. |
|  |  | (b) Indicate number of participants with missing data for each variable of interest | Table 1 |
|  |  | (c) Summarise follow-up time (eg, average and total amount) | Information was available for up to 30 days for all included patients. |
| **Outcome data** | 15* | Report numbers of outcome events or summary measures over time | Included in Results (Results section, Tables 1 – 3) |
| **Main results** | 16 | (*a*) Give unadjusted estimates and, if applicable, confounder-adjusted estimates and their precision (eg, 95% confidence interval). Make clear which confounders were adjusted for and why they were included | Specified in Results (Tables 1-3) |
|  |  | (*b*) Report category boundaries when continuous variables were categorized | Continuous variables were not categorized. |
|  |  | (*c*) If relevant, consider translating estimates of relative risk into absolute risk for a meaningful time period | Not applicable. |
| **Other analyses** | 17 | Report other analyses done—eg analyses of subgroups and interactions, and sensitivity analyses | Reported in Methods. |
| Discussion | | |  |
| **Key results** | 18 | Summarise key results with reference to study objectives | Specified in Abstract and Discussion. |
| **Limitations** | 19 | Discuss limitations of the study, taking into account sources of potential bias or imprecision. Discuss both direction and magnitude of any potential bias | Included in Discussion. |
| **Interpretation** | 20 | Give a cautious overall interpretation of results considering objectives, limitations, multiplicity of analyses, results from similar studies, and other relevant evidence | Explained in Discussion. |
| **Generalisability** | 21 | Discuss the generalisability (external validity) of the study results | Included in Discussion. |
| Other information | | |  |
| Funding | 22 | Give the source of funding and the role of the funders for the present study and, if applicable, for the original study on which the present article is based | Included. |
