# Supplementary material for: Inpatient versus outpatient management of community-acquired acute skin and soft tissue infections. Clinical outcomes and factors associated with eligibility for early discharge
Source: BMC Infect Dis. 2025 Nov 17;25:1594. doi: 10.1186/s12879-025-11883-6 (PMC12625354; doi:10.1186/s12879-025-11883-6)
Supplement: Supplementary file 2 — Supplementary Material 2. [file 12879_2025_11883_MOESM2_ESM.docx]

| Supplementary Table S2. Univariate and multivariate analysis of parameters predicting recurrence. | | | | | | | |
| --- | --- | --- | --- | --- | --- | --- | --- |
|  | **Recurrence cohort sample (n=315)** | | | | | | |
|  | **Recurrence**  **(n =31)** | **Non-Recurrence**  **(n =284)** | **Unadjusted OR**  **(95% CI)** | ***p-value*** | | **Adjusted OR**  **(95% CI)** | ***p-value*** |
| Management  Outpatient  Hospitalization | 12 (7.5)  19 (12.3) | 148 (92.5)  136 (87.7) | -  1.72 (0.81-3.68) |  | -  0.163 | 1.00  0.97 (0.41-2.28) | 0.943 |
| Demographics and social conditions  Age (years), m (IQR) | 49 (43.5-64.5) | 59 (43.8-75.2) | 0.99 (0.97-1.01) |  | 0.372 | 1.00 (0.97-1.02) | 0.803 |
| Male sex  Female sex | 21 (10.7)  10 (8.40) | 175 (89.3)  109 (91.6) | -  0.76 (035-1.68) |  | -  0.518 | 1.00  0.90 (0.38-2.14) | 0.810 |
| History of drug injection  Homelessness  Social and economic barriers to care | 8 (26.7)  7 (20)  5 (12.8) | 22 (73.3)  28 (80)  34 (87.2) | 4.14 (1.66-10.3)  2.67 (1.05-6.74)  1.41 (0.51-3.93) |  | **0.005**  0.054  0.501 | 2.02 (0.67-6.07) | 0.209 |
|  |  |  |  |  |  |  |  |
| Underlying conditions |  |  |  |  |  |  |  |
| Charlson Comorbidity Index,  m(IQR) | 2 (0-4) | 2 (0-5) | 1.00 (0.87-1.14) |  | 0.964 |  |  |
| Diabetes mellitus | 7 (9.59) | 66 (90.4) | 0.96 (0.40-2.34) |  | 0.960 |  |  |
| COPD | 3 (17.6) | 14 (82.4) | 2.07 (0.56-7.63) |  | 0.301 |  |  |
| Congestive heart failure | 1 (4.35) | 22 (95.7) | 0.40 (0.05-3.05) |  | 0.397 |  |  |
| Cirrhosis | 5 (38.5) | 8 (61.5) | 6.63 (2.02-21.8) |  | **0.005** |  |  |
| Neurological disorder  Chronic kidney disease | 4 (16)  2 (5.88) | 21 (84)  32 (94.1) | 1.86 (0.59-5.80)  0.54 (0.12-2.38) |  | 0.306  0.446 |  |  |
| Hematologic malignancy | 0 (0) | 5 (100) | 0.00 (0.00;.) |  | 0.594 |  |  |
| Solid tumor malignancy  Mental illness  Immunosuppression  HIV/AIDS  Kidney transplant | 3 (15.8)  1 (5.88)  2 (10.5)  0 (0) | 16 (84.2)  16 (94.1)  17 (89.5)  7 (100) | 1.79 (0.49-6.54)  0.56 (0.07-4.36)  1.08 (0.24-4.93)  0.00 (0.00;.) |  | 0.388  0.649  0.862  0.481 |  |  |
|  |  |  |  |  |  |  |  |
| SSTI classification |  |  |  |  |  |  |  |
| Cellulitis / Erysipelas | 26 (10.2) | 228 (89.8) | - |  | - |  |  |
| Surgical or traumatic wound infection | 2 (33.3) | 4 (66.7) | 4.38 (0.77-25.1) |  | 0.146 |  |  |
| Skin abscess | 2 (3.77) | 51 (96.2) | 0.34 (0.08-1.50) |  | 0.133 |  |  |
| Necrotizing fasciitis | 1 (50) | 1 (50) | 8.77 (0.53- 144) |  | 0.211 |  |  |
|  |  |  |  |  |  |  |  |
| Localization  Lower extremities  Upper extremities  Other  Various localizations | 29 (11.8)  1 (3.33)  1 (3.03)  0 (0) | 217 (88.2)  29 (96.7)  32 (97)  6 (100) | -  0.26 (0.03-1.97)  0.23 (0.03-1.78)  0.00 (0.00;.) |  | -  0.158  0.120  0.476 |  |  |
| Portal of entry  Surgical or traumatic wound  Ulcer  Fungal infection  Skin lesion  Others  Unknown | 9 (10.7)  10 (20.8)  4 (17.4)  0 (0)  0 (0)  8 (6.96) | 75 (89.3)  38 (79.2)  19 (82.6)  19 (100)  26 (100)  107 (93) | -  2.19 (0.82-5.85)  1.75 (0.49-6.32)  0.00 (0.00;.)  0.00 (0.00;.)  0.62 (0.23-1.69) |  | **-**  0.126  0.405  0.079  0.147  0.364 |  |  |
| Predisposing factors  None  One or more factors | 6 (4.65)  25 (13.4) | 123 (95.3)  161 (88.6) | -  3.18 (1.27-8.00) |  | -  **0.009** |  |  |
| Previous episodes of SSTIs  Recurrent SSTIs | 15 (20)  16 (28.1) | 60 (80)  41 (71.9) | 3.50 (1.64-7.48)  6.32 (2.90-13.8) |  | **0.002**  **<0.001** | 5.86 (2.51-13.67) | **<0.001** |
|  |  |  |  |  |  |  |  |
| Hospital-at-home  Voluntary discharge | 1 (5.26)  3 (15.8) | 18 (94.7)  16 (84.2) | 0.49 (0.06-3.82)  1.79 (0.49-6.54) |  | 0.553  0.388 |  |  |
|  |  |  |  |  |  |  |  |
| Baseline illness severity |  |  |  |  |  |  |  |
| SOFA score, m (IQR) | 0 (0-1) | 0 (0-1) | 1.28 (1.03-1.59) |  | **0.028** | 1.21 (0.93-1.58) | 0.153 |
| SAPS II | 24 (19-32.5) | 24 (19-30) | 1.01 (0.97-1.06) |  | 0.489 |  |  |
| Sepsis | 1 (25) | 3 (75) | 3.29 (0.33-32.7) |  | 0.374 |  |  |
| Septic shock | 2 (28.6) | 5 (71.4) | 3.94 (0.73-21.3) |  | 0.161 |  |  |
| ICU admission | 1 (20) | 4 (80) | 2.33 (0.25-21.6) |  | 0.484 |  |  |
| Bacteremia | 2 (14.3) | 12 (85.7) | 1.56 (0.33-7.33) |  | 0.558 |  |  |
|  |  |  |  |  |  |  |  |
| Therapeutic management |  |  |  |  |  |  |  |
| Appropriate treatment | 7 (10) | 63 (90) | 1.02 (0.42-2.47) |  | 0.943 |  |  |
| 72h delay in initiating appropriate antibiotic therapy | 1 (20) | 4 (80) | 2.50 (0.24-26.1) |  | 0.487 |  |  |
|  |  |  |  |  |  |  |  |
| Source control  Not required  Surgery  Percutaneous drainage | 28 (10.6)  3 (11.1)  0 (0) | 236 (89.4)  24 (88.9)  23 (100) | -  1.05 (0.30-3.72)  0.00 (0.00;. |  | -  0.890  0.085 |  |  |

Data are presented as nos. (%) unless otherwise specified. Abbreviations: AIDS (acquired immunodeficiency syndrome), COPD (chronic obstructive pulmonary disease), ED (emergency department), HIV (human immunodeficiency virus), ICU (intensive care unit), IQR (interquartile range), m (median), SAPS II (Simplified Acute Physiology Score), SOFA (Sequential Organ Failure Assessment), SSTI(s) (skin and soft tissue infection(s)).
