# Supplementary material for: Inpatient versus outpatient management of community-acquired acute skin and soft tissue infections. Clinical outcomes and factors associated with eligibility for early discharge
Source: BMC Infect Dis. 2025 Nov 17;25:1594. doi: 10.1186/s12879-025-11883-6 (PMC12625354; doi:10.1186/s12879-025-11883-6)
Supplement: Supplementary file 3 — Supplementary Material 3. [file 12879_2025_11883_MOESM3_ESM.docx]

| Suplementary Table S3. Univariate and multivariate analysis of parameters predicting unplanned readmissions related to the SSTI. | | | | | | | |
| --- | --- | --- | --- | --- | --- | --- | --- |
|  | **Unplanned readmissions recurrence sample (n=319)** | | | | | | |
|  | **Unplanned readmissions**  **(n =53)** | **Non-Unplanned readmissions**  **(n =266)** | **Unadjusted OR**  **(95% CI)** | ***p- value*** | | **Adjusted OR**  **(95% CI)** | ***p-value*** |
| Management  Outpatient  Hospitalization | 26 (16.2)  27 (17) | 134 (83.3)  132 (83) | -  1.05 (0.58-1.90) |  | -  0.862 | 1.00  0.89 (0.48-1.67) | 0.724 |
| Demographics and social conditions  Age (years), m (IQR) | 64 (45-76) | 57 (43-72.5) | 1.01 (1.00-1.03) |  | 0.091 | 1.01 (0.99-1.03) | 0.342 |
| Male sex  Female sex | 38 (19)  15 (12.6) | 162 (81)  104 (87.4) | -  0.61 (0.32-1.17) |  | -  0.139 | 1.00  0.58 (0.29-1.16) | 0.124 |
| History of drug injection  Homelessness  Social and economic barriers to care | 4 (12.5)  8 (21.1)  6 (14) | 28 (87.5)  30 (78.9)  37 (86) | 0.69 (0.23-2.07)  1.40 (0.60-3.25)  0.79 (0.32-1.98) |  | 0.540  0.436  0.642 | 0.49 (0.13-1.87)  2.15 (0.77-6.02) | 0.294  0.146 |
|  |  |  |  |  |  |  |  |
| Underlying conditions |  |  |  |  |  |  |  |
| Charlson Comorbidity Index,  m(IQR) | 4 (1-6) | 2 (0-4) | 1.15 (1.04-1.26) |  | **0.006** |  |  |
| Diabetes mellitus | 19 (26) | 54 (74) | 2.19 (1.16-4.14) |  | **0.019** | 2.04 (1.01-4.10) | **0.046** |
| COPD | 7 (41.2) | 10 (58.8) | 3.90 (1.41-10.8) |  | 0.015 |  |  |
| Congestive heart failure | 2 (8.70) | 21 (91.3) | 0.46 (0.10-2.01) |  | 0.307 |  |  |
| Cirrhosis | 5 (35.7) | 9 (64.3) | 2.97 (0.96-9.26) |  | 0.080 |  |  |
| Neurological disorder  Chronic kidney disease | 7 (28)  9 (26.5) | 18 (72)  25 (73.5) | 2.10 (0.83-5.30)  1.97 (0.86-4.51) |  | 0.136  0.123 |  |  |
| Hematologic malignancy | 1 (20) | 4 (80) | 1.26 (0.14-11.5) |  | 0.794 |  |  |
| Solid tumor malignancy  Mental illness  Immunosuppression  HIV/AIDS  Kidney transplant | 3 (15.8)  0 (0)  1 (4.55)  2 (28.6) | 16 (84.2)  17 (100)  21 (95.5)  5 (71.4) | 0.94 (0.26-3.34)  0.00 (0.00;.)  0.22 (0.03-1.71)  2.05 (0.39-10.8) |  | 0.969  **0.042**  0.107  0.422 |  |  |
|  |  |  |  |  |  |  |  |
| SSTI classification |  |  |  |  |  |  |  |
| Cellulitis / Erysipelas | 47 (18.3) | 210 (81.7) | - |  | - |  |  |
| Surgical or traumatic wound infection | 2 (33.3) | 4 (66.7) | 2.23 (0.40-12.6) |  | 0.391 |  |  |
| Skin abscess | 3 (5.56) | 51 (94.4) | 0.26 (0.08-0.88) |  | **0.014** |  |  |
| Necrotizing fasciitis | 1 (50) | 1 (50) | 4.47 (0.27-72.7) |  | 0.371 |  |  |
|  |  |  |  |  |  |  |  |
| Localization  Lower extremities  Upper extremities  Other  Various localizations | 47 (19)  3 (9.38)  3 (9.09)  0 (0) | 201 (81)  29 (90.6)  30 (90.9)  6 (100) | -  0.44 (0.13-1.51)  0.43 (0.13-1.46)  0.00 (0.00;.) |  | -  0.163  0.163  0.289 |  |  |
| Portal of entry  Surgical or traumatic wound  Ulcer  Fungal infection  Skin lesion  Others  Unknown | 16 (18.2)  13 (27.1)  4 (17.4)  1 (5.26)  2 (7.69)  17 (14.8) | 72 (81.8)  35 (72.9)  19 (82.6)  18 (97.4)  24 (92.3)  98 (85.2) | -  1.67 (0.72-3.86)  0.95 (0.28-3.17)  0.25 (0.03-2.01)  2.38 (0.08-1.75)  0.78 (0.37-1.65) |  | **-**  0.238  0.961  0.171  0.209  0.521 |  |  |
| Predisposing factors  None  One or more factors | 14 (10.8)  39 (20.6) | 116 (89.2)  150 (79.4) | -  2.15 (1.12-4.16) |  | **-**  **0.019** |  |  |
| Previous episodes of SSTIs  Recurrent SSTIs | 17 (22.7)  18 (31.6) | 58 (77.3)  39 (68.4) | 1.69 (0.89-3.23)  2.99 (1.54-5.80) |  | 0.119  **0.002** |  |  |
|  |  |  |  |  |  |  |  |
| Hospital-at-home  Voluntary discharge | 3 (15.8)  4 (17.4) | 16 (84.2)  19 (82.6) | 0.94 (0.26-3.34)  1.06 (0.35-3.26) |  | 0.969  0.881 |  |  |
|  |  |  |  |  |  |  |  |
| Baseline illness severity |  |  |  |  |  |  |  |
| SOFA score, m (IQR) | 0 (0-1) | 0 (0-1) | 1.28 (1.05-1.56) |  | **0.013** | 1.25 (1.01-1.53) | **0.039** |
| SAPS II | 27 (19-32) | 24 (19-30) | 1.04 (1.01-1.08) |  | **0.011** |  |  |
| Sepsis | 1 (25) | 3 (75) | 1.72 (0.18-16.9) |  | 0.639 |  |  |
| Septic shock | 2 (28.6) | 5 (71.4) | 2.06 (0.39-10.9) |  | 0.417 |  |  |
| ICU admission | 1 (20) | 4 (80) | 1.26 (0.14-11.5) |  | 0.794 |  |  |
| Bacteremia | 3 (21.4) | 11 (78.6) | 1.39 (0.37-5.17) |  | 0.607 |  |  |
|  |  |  |  |  |  |  |  |
| Therapeutic management |  |  |  |  |  |  |  |
| Appropriate treatment | 12 (17.1) | 58 (82.9) | 1.04 (0.52-2.12) |  | 0.888 |  |  |
| 72h delay in initiating appropriate antibiotic therapy | 1 (20) | 4 (80) | 1.40 (0.14-13.9) |  | 0.749 |  |  |
|  |  |  |  |  |  |  |  |
| Source control  Not required  Surgery  Percutaneous drainage | 50 (18.7)  2 (7.41)  1 (4.35) | 217 (81.3)  25 (92.6)  22 (95.7) | -  0.35 (0.08-1.51)  0.20 (0.03-1.50) |  | -  0.138  0.071 |  |  |

Data are presented as nos. (%) unless otherwise specified. Abbreviations: AIDS (acquired immunodeficiency syndrome), COPD (chronic obstructive pulmonary disease), ED (emergency department), HIV (human immunodeficiency virus), ICU (intensive care unit), IQR (interquartile range), m (median), SAPS II (Simplified Acute Physiology Score), SOFA (Sequential Organ Failure Assessment), SSTI(s) (skin and soft tissue infection(s)).
