# Supplementary material for: Inpatient versus outpatient management of community-acquired acute skin and soft tissue infections. Clinical outcomes and factors associated with eligibility for early discharge
Source: BMC Infect Dis. 2025 Nov 17;25:1594. doi: 10.1186/s12879-025-11883-6 (PMC12625354; doi:10.1186/s12879-025-11883-6)
Supplement: Supplementary file 4 — Supplementary Material 4. [file 12879_2025_11883_MOESM4_ESM.docx]

| Suplementary Table S4. Univariate and multivariate analysis of parameters predicting ED visits related to the SSTI. | | | | | | | |
| --- | --- | --- | --- | --- | --- | --- | --- |
|  | **ED visits recurrence sample (n =320)** | | | | | | |
|  | **ED visits**  **(n =69)** | **Non-ED visits**  **(n =251)** | **Unadjusted OR**  **(95% CI)** | ***p-value*** | | **Adjusted OR**  **(95% CI)** | ***p-value*** |
| Management  Outpatient  Hospitalization | 41 (25.6)  28 (17.5) | 119 (74.4)  132 (82.5) | -  0.62 (0.36-1.06) |  | -  0.079 | 1.00  0.32 (0.16-0.62) | **0.001** |
| Demographics and social conditions  Age (years), m (IQR) | 55 (43-70) | 59 (43.5-74) | 1.00 (0.98-1.01) |  | 0.531 | 1.01 (0.99-1.03) | 0.274 |
| Male sex  Female sex | 50 (25)  19 (15.8) | 150 (75)  101 (84.2) | -  0.56 (0.31-1.01) |  | -  0.053 | 1.00  0.60 (0.32-1.14) | 0.119 |
| History of drug injection  Homelessness  Social and economic barriers to care | 12 (37.5)  15 (38.5)  14 (32.6) | 20 (62.5)  24 (61.5)  29 (67.4) | 2.43 (1.12-5.26)  2.63 (1.29-5.34)  1.95 (0.96-3.94) |  | **0.031**  **0.010**  0.072 | 1.00 (0.36-2.78)  3.35 (1.32-8.46) | 0.996  **0.011** |
|  |  |  |  |  |  |  |  |
| Underlying conditions |  |  |  |  |  |  |  |
| Charlson Comorbidity Index,  m(IQR) | 2 (0-4) | 2 (0-5) | 0.97 (0.88-1.07) |  | 0.512 |  |  |
| Diabetes mellitus | 17 (23.3) | 56 (76.7) | 1.4 (0.61-2.12) |  | 0.678 |  |  |
| COPD | 4 (23.5) | 13 (76.5) | 1.13 (0.36-3.57) |  | 0.812 |  |  |
| Congestive heart failure | 3 (13) | 20 (87) | 0.52 (0.15-1.82) |  | 0.318 |  |  |
| Cirrhosis | 5 (35.7) | 9 (64.3) | 2.10 (0.68-6.49) |  | 0.218 |  |  |
| Neurological disorder  Chronic kidney disease | 7 (28)  7 (20.6) | 18 (72)  27 (79.4) | 1.46 (0.58-3.66)  0.94 (0.39-2.25) |  | 0.421  0.911 |  |  |
| Hematologic malignancy | 1 (20) | 4 (80) | 0.91 (0.10-8.26) |  | 1.000 |  |  |
| Solid tumor malignancy  Mental illness  Immunosuppression  HIV/AIDS  Kidney transplant | 2 (10.5)  5 (29.4)  3 (13.6)  0 (0) | 17 (89.5)  12 (70.6)  19 (86.4)  7 (100) | 0.41 (0.09-1.82)  1.56 (0.53-4.58)  0.56 (0.16-1.93)  0.00 (0.00;.) |  | 0.238  0.428  0.370  0.179 |  |  |
|  |  |  |  |  |  |  |  |
| SSTI classification |  |  |  |  |  |  |  |
| Cellulitis / Erysipelas | 58 (22.5) | 200 (77.5) | - |  | - |  |  |
| Surgical or traumatic wound infection | 2 (33.3) | 4 (66.7) | 1.72 (0.21-9.65) |  | 0.545 |  |  |
| Skin abscess | 8 (14.8) | 46 (85.2) | 0.60 (0.27-1.34) |  | 0.212 |  |  |
| Necrotizing fasciitis | 1 (50) | 1 (50) | 3.45 (0.21-56) |  | 0.454 |  |  |
|  |  |  |  |  |  |  |  |
| Localization  Upper extremities  Lower extremities  Other  Various localizations | 5 (15.6)  57 (22.9)  7 (21.2)  0 (100) | 27 (84.4)  192 (77.1)  26 (76.8)  6 (100) | 0.62 (0.23-1.69)  -  0.91 (0.37-2.20)  0.00 (0.00;.) |  | 0.366  -  0.855  0.215 |  |  |
| Portal of entry  Surgical or traumatic wound  Ulcer  Fungal infection  Skin lesion  Others  Unknown | 20 (22.7)  12 (25)  4 (17.4)  17 (89.5)  7 (26.9)  24 (20.7) | 68 (77.3)  36 (75)  19 (82.6)  2 (10.5)  19 (73.1)  92 (79.3) | -  1.13 (0.50-2.58)  0.72 (0.22-2.35)  0.40 (0.09-1.88)  1.25 (0.46-3.40)  0.89 (0.45-1.74) |  | **-**  0.763  0.610  0.249  0.656  0.727 |  |  |
| Predisposing factors  None  One or more factors | 29 (22.3)  40 (21.1) | 101 (77.7)  150 (78.9) | -  0.93 (0.54-1.59) |  | **-**  0.787 |  |  |
| Previous episodes of SSTIs  Recurrent SSTIs | 22 (29.3)  25 (43.9) | 53 (70.7)  32 (56.1) | 1.75 (0.97-3.15)  3.89 (2.10-7.19) |  | 0.069  **<0.001** | 6.47 (3.05-13.71) | **<0.001** |
|  |  |  |  |  |  |  |  |
| Hospital-at-home  Voluntary discharge | 3 (15.8)  10 (43.5) | 16 (84.2)  13 (56.5) | 0.67 (0.19-2.36)  3-10 (1.30-7.42) |  | **0.565**  **0.015** |  |  |
|  |  |  |  |  |  |  |  |
| Baseline illness severity |  |  |  |  |  |  |  |
| SOFA score, m (IQR) | 0 (0-1) | 0 (0-0) | 1.11 (0.91-1.35) |  | 0.294 |  |  |
| SAPS II | 24 (19-30) | 24 (19-30) | 1.00 (0.97-1.03) |  | 0.890 |  |  |
| Sepsis | 1 (25) | 3 (75) | 1.23 (0.13-12) |  | 0.822 |  |  |
| Septic shock | 2 (28.6) | 5 (71.4) | 1.47 (0.28-7.76) |  | 0.639 |  |  |
| ICU admission | 1 (20) | 4 (80) | 0.91 (0.10-8.26) |  | 1.000 |  |  |
| Bacteremia | 5 (35.7) | 9 (64.3) | 2.10 (0.68-6.49) |  | 0.218 |  |  |
|  |  |  |  |  |  |  |  |
| Therapeutic management |  |  |  |  |  |  |  |
| Appropriate treatment | 13 (18.6) | 57 (81.4) | 0.80 (0.41-1.58) |  | 0.538 |  |  |
| 72h delay in initiating appropriate antibiotic therapy | 2 (40) | 3 (60) | 3.73 (0.55-25.3) |  | 0.227 |  |  |
|  |  |  |  |  |  |  |  |
| Source control  Not required  Surgery  Percutaneous drainage | 60 (22.4)  4 (14.8)  4 (17.4) | 208 (77.6)  23 (85.2)  19 (82.6) | -  0.60 (0.20-1.81)  0.73 (0.24-2.23) |  | -  0.381  0.612 |  |  |

Data are presented as nos. (%) unless otherwise specified. Abbreviations: AIDS (acquired immunodeficiency syndrome), COPD (chronic obstructive pulmonary disease), ED (emergency department), HIV (human immunodeficiency virus), ICU (intensive care unit), IQR (interquartile range), m (median), SAPS II (Simplified Acute Physiology Score), SOFA (Sequential Organ Failure Assessment), SSTI(s) (skin and soft tissue infection(s)).
